# The “Criminal Shield”: Criminal Liability for Healthcare Professionals during the COVID-19 Pandemic

**DOI:** 10.3390/healthcare11192661

**Published:** 2023-10-01

**Authors:** Giorgio Bolino, Gianpiero D’Antonio, Letizia Sorace, Nicola Di Fazio, Gianpietro Volonnino, Raffaele La Russa, Mauro Arcangeli, Paola Frati

**Affiliations:** 1 Department of Anatomical, Histological, Forensic and Orthopedic Sciences, Sapienza University of Rome, 00128 Rome, Italy; giorgio.bolino@uniroma1.it (G.B.); gianpiero.dantonio@uniroma1.it (G.D.); letizia.sorace@uniroma1.it (L.S.); gianpietro.volonnino@uniroma1.it (G.V.); paola.frati@uniroma1.it (P.F.); 2 Department of Clinical and Experimental Medicine, University of Foggia, 71122 Foggia, Italy; raffaele.larussa@unifg.it; 3 Department of Life, Health and Environmental Sciences, University of L’Aquila, 67100 L’Aquila, Italy; mauro.arcangeli@uniaq.it

**Keywords:** liability, COVID-19 vaccination, ethical issues

## Abstract

The Sars-CoV-2 pandemic has had important economic, health, political, and jurisprudential implications all over the world. According to innovations already introduced by Law 24/2017, with Decree Law no. 44 of 1 April 2021 and the subsequent conversion law no. 71 of 2021, Italy is the only country in which ad hoc rules have been introduced to limit the professional liability of healthcare professionals during the health emergency. The “criminal shield” can be defined as the Legislator response to the extreme pressure on healthcare professionals during the pandemic.

## 1. Introduction

After the outbreak of the SARS-CoV strain in China (province of Hubei) in March 2020, a state of pandemic was declared by the World Health Organization. This brought significant consequences all over the world from a health, economic, political, and juridical point of view [1]. New specific vaccines were introduced on the market to fight the spread of SARS-CoV-2 and strong awareness campaigns were made worldwide. In Italy, as well as in Europe, the vaccination campaign began at the end of December 2020 with the so-called “vaccination days”, followed by the introduction of the European Digital COVID Certificate, generally known as “Green Pass” [2,3]. A variety of vaccines were introduced on the market, each of which use different technologies such as: mRNA vaccines (Pfizer/BioNTech and Moderna), viral vector vaccines (Vaxzervria ex AstraZeneca and Janssen of the Johnson & Johnson group) and the most recent Nuvaxovid (Novavax), based on recombinant DNA technology. The efficacy and safety of the inoculation of these vaccinations has been scientifically well examined by the scientific community, which is able to affirm that the level of risk is absolutely commensurate and acceptable [4,5]. Though, ambiguities about vaccination against COVID-19 still persist. This was due to the media overexposure of the phenomenon as well as the mandatory vaccination for some categories of the population such as healthcare workers. The duration of the immunity produced by the vaccine, its difference with the one induced by the contraction of the infection, the real possibility of reducing the spread of the virus, the efficacy in different kinds of populations, and the general safety for mainly children and pregnant women are some of the questions and doubts that have plagued both vaccinating doctors and the general population. Those doubts slowed down the vaccination campaign in Italy. In addition, the Italian government has regulated by law for the COVID-19 vaccination of incapacitated subjects admitted to assisted health facilities [6].

Obviously, the field of professional liability was also affected to some extent by the pandemic. In the exercise of professional activity, all physicians, thus including those engaged in vaccination activities, are liable for the consequences arising from their own unlawful conduct (commissive or omissive) in violation of a rule. This is valid in the criminal, civil, disciplinary, and treasury spheres.

In criminal proceedings, liability is personal, pursuant to Article 27, Paragraph 1 of the Italian Constitution. Therefore, the physician who is attributed the authorship of the offence is personally liable for the consequences of the wrongdoing. In Italy, thanks to Law No. 24/2017 [7] (“Gelli-Bianco Law”), the article 590-sexies was introduced into the Penal Code. The Gelli-Bianco Law aims at ensuring the safety of care, which is defined as the specific process that aims to avoid, prevent, and mitigate adverse effects or harm resulting from healthcare provisions. In this context, the article 590-sexies establishes a cause of non-punishability for the physician or the healthcare professional (e.g., physicians, nurses, physiotherapists, and midwifes) when his or her unskillful conduct causes the death or personal injury of the patient, only if the accredited guidelines provided by the Italian National Institute of Health have been respected and that they were appropriate to the specific case. Unskillfulness is defined as inability, incompetence, unpreparedness, and/or the lack of specific skills in one’s profession. On the contrary, the healthcare professional is punishable in the case of an event which is caused by negligence or imprudence (even in the configuration of slight culpability). Furthermore, the healthcare professional is still punishable if the gross culpability is caused by unskillfulness or if the guidelines are unsuitable for the specific case. The conspicuous number of deaths that have occurred and the consequent risk of litigation for healthcare professionals have made it necessary to introduce ad hoc rules [8]. With a pandemic caused by a new pathogen, such as SARS-CoV-2, with the absence of adequate resources and scientific knowledge, the regulation provided by Law 24/2017 was unsuitable. This can be outlined, for example, by the compassionate use (especially in the early stages of the pandemic) of drugs still in the testing phase (e.g., Remdesivir) [9], as well as the use of off-label drugs (cf. AIFA Circular of April the sixth 2020; e.g., hydroxychloroquine). Additionally, factors that characterized the state of the pandemic, such as limited resources and the extraordinary and exhausting rhythms sustained by healthcare workers, with a significant increase in psychophysical stress conditions, inevitably lead to increases in episodes of negligence and imprudence [10,11,12]. For instance, it does not appear to be a coincidence that with Decree Law no. 8 of April the 2nd 2020, medicine and surgery degree courses became “enabling” for the profession. As a result, that measure led to the employment of thousands of new physicians. 

While other jurisprudential and medico-legal areas have already been well examined [13], this communication paper aims to present the Italian situation regarding the professional liability of healthcare professionals from a criminal point of view in the context of the COVID-19 pandemic.

## 2. Italian Context in Professional Liability during the Pandemic: An Overview in Criminal Matters

Italy is the only main European country where a new law has been introduced in order to define the limits of professional liability in criminal matters during the pandemic [14].

In fact, regarding the subject of criminal liability, with article 3 of Decree Law no. 44 of 1 April 2021, it was established that: “ for the events referred to in Articles 589 and 590 of the Criminal Code that occurred due to the administration of a vaccine for the prevention of Sars-cov-2 infections, carried out during the extraordinary vaccination campaign, in implementation of the plan outlined in article 1, paraghraph 457, of the Law of 30 December 2020, no 178, punishability is excluded when the use of the vaccine complies with the indications contained in the marketing authorization measure issued by the competent authorities and in the circulars published on institutional site of the Ministry of Health related to vaccination activity”.

In this sense, concerning the deaths that followed, at least chronologically, some administrations of the viral vector vaccine ex AstraZeneca (now VaxZevria) [15] were very significant. As shown in the Explanatory Report to Decree Law no. 44 of 2021, this intervention had the goal of “reassuring healthcare personnel and in general those involved in vaccination activities” about the fear that defensive medicine attitudes could cause repercussions on the timing of the administration of the vaccination, which at the time represented one of the most effective measures to control the pandemic [16].

Subsequently, with the conversion law no. 71 of 2021, in force since June 1 of the same year, the Parliament, incorporating the complaints of professional associations (e.g., FNOMCeO) [17] expanded the possibility of the limitation of culpable liability for death or personal injury in the health care sector during the state of emergency from COVID-19. The text of the aforementioned law reads as follows: “During the state of epidemiological emergency from COVID 19, declared by a resolution of the Council of Ministers of 31 January 2020, and subsequent extensions, the facts referred to in Articles 589 and 590 of the Criminal Code, committed in the exercise of a health profession and which are caused by the emergency situation, are punishable only in cases of gross culpability. 2 For the purpose of assessing the degree of culpability, the court shall take into account, among the factors that may exclude its seriousness, the limited scientific knowledge at the time of the act on SARS-CoV-2 diseases and appropriate treatment, the scarcity of human and material resources concretely available in relation to the number of cases to be treated, as well as the lower degree of experience and technical knowledge possessed by the non-specialized personnel employed to deal with the emergency”.

The “criminal shield” previously introduced with art. 3 of Decree Law no. 44 of 1 April 2021 was originally limited only to the vaccination area and was therefore extended by providing a limitation of criminal liability for the cases of gross culpability for all the healthcare professionals during the emergency. 

## 3. Discussion

By Decree Law no. 44 of 1 April 2022 and the conversion law no. 71 of 2021, more favorable criminal rules were introduced compared to art. 590-sexies, in paragraph 2 of the Criminal Code. Those rules are applied retroactively (i.e., from January the thirty-first 2020 to July the thirty-first 2021), pursuant to and for the purposes of articles 3 of the Constitution and 2, in paragraph 4 of the Criminal Code.

These rules, in addition to being intended as the Legislature’s response to the issue of health care liability for adverse events occurring during the emergency period, were part of a broader debate about the suitability of coining special “shield rules” for health care personnel that were not present until then.

The two, which are temporary and limited to the emergency phase, are thus an expression of two different regulatory models: 1. Art. 3, limited to vaccination activity, which corresponds to the total exemption of criminal responsibility (without any reference to the degree of guilt), and 2. art. 3 bis, extended to all health activities related to the state of emergency that lies within the scheme of reparameterization of criminal liability, with the exclusion of cases of gross culpability.

Therefore, in line with similar initiatives aimed at limiting medical–legal litigation against healthcare professionals in the international scene [18], the exemption of criminal (but not civil) liability for vaccinating doctors, pursuant to Article 3 of Decree Law 44 of 2021, converted without amendments, is provided in case three requirements subsist: the occurrence of death or personal injury of the vaccinated person, the existence of a causal relationship between the vaccine and adverse events, and the compliance of vaccine administration with precautionary rules. The latter guidance is provided in the marketing authorization order issued by the competent authorities and in circulars published on the institutional website of the Ministry of Health regarding vaccination activity. As an example, AstraZeneca updated the SmPC (summary of product characteristics) and package leaflet of Vaxzevria (on 4 July 2021) with information on the cases of disseminated intravascular coagulation and cerebral venous sinus thrombosis that occurred [19]. In this case, the physician should be familiar with the contents of the latest update and related ministerial circulars.

Obviously, the ascertainment of the causal relationship between damage and vaccination must take place in the trial, having operational repercussions in terms of *notitia criminis*. In other words, in the procedural field, the investigative authorities should no longer proceed against the healthcare professional but rather against an unknown person.

The operational area is related to the actual vaccine administration and to its prodromal phase (pre-vaccination triage and anamnestic questionnaire). It is precisely in the preliminary phase, rather than the inoculation one characterized by the simple execution of an intramuscular injection, that the most critical issues, from a medical–legal point of view, may arise.

The collection of informed consent that precedes the administration of the vaccine is, in fact, one of the key moments of the vaccinating physician’s medical–legal responsibility. As a matter of fact, the doctor during the anamnestic phase assesses and analyzes the suitability of the patient for vaccination, explains the procedure and adverse events, and exposes the risks and benefits. Thus, as defined by paragraph 8 of Article 1 of Law 219/2017 (“The time of communication between doctor and patient is time of care”) [20], a moment of care is realized. Certainly, in order for the consent to be truly informed, it is necessary for the vaccinating physician to be constantly updated, given the continuous implementation of knowledge about the pandemic, Sars-CoV-2-related diseases, available vaccines, and their indications.

Article 3-bis of Decree Law no. 44 of 2021 defines the cause of non-punishability of non-gross culpability for facts causally attributable to the Covid-19 pandemic arising not only from inexperience but also from negligence and imprudence, ensuring judicial protection in the cases of willful misconduct or gross culpability (therefore not justifiable even in an emergency context). Thus, article 3-bis is limited to the facts exclusively framed as manslaughter and culpable personal injury (articles 589 and 590 of the Criminal Code), excluding other cases such as, for example, culpable epidemic crimes (articles 438 and 452 of the Criminal Code). 

The crimes not punishable under Article 3-bis have to be related to the “health emergency situation” from Sars-CoV2. This implies that, for the purposes of exemption, it is not enough that the acts happened during the state of emergency deliberated by the government, but must have occurred in a situation of emergency, i.e., clinical impellency, such that the normal clinical–diagnostic and therapeutic process is altered.

The provision under the analysis emphasizes the temporary, as well as the exceptional nature, of the state of emergency. Nevertheless, it should be specified that events causally linked to the emergency, though occurring at a later time, are included in the cases provided for by law. 

Moreover, article 3 bis gives indications regarding the judges’ assessment tools. For the purpose of assessing the degree of guilt, it will certainly be necessary to consider the limited scientific knowledge at the time of the fact about the diseases related to SARS-CoV2 (considering both the pathological findings and the appropriate therapies), the scarcity of human and material resources concretely available during an emergency situation, and the lower degree of experience and technical knowledge possessed by the non-specialized personnel employed to cope with the emergency.

## 4. Conclusions

Following the medical liability revolution initiated by L. 24/2017, the pandemic experience and the persistent debate about the enactment of ad hoc laws have been particularly relevant in terms of professional liability. As a matter of fact, considering the first important steps dictated by the introduction of the Decree Law no. 44 of 1 April 2021 and the conversion law no. 71 of 2021, which straiten the criminal liability of healthcare professionals during the COVID pandemic and the related vaccination campaign, the pandemic itself can be the primum movens of a wider process of improvement of the current legislative statute to protect both healthcare professionals and patients.

In conclusion, as previously described, this “criminal shield” has been the Legislator’s response to the issue of healthcare liability in Italy during the pandemic. This can be considered an embraceable initiative by all healthcare professionals. This applies both in the context of the COVID-19 pandemic, during which healthcare professionals have been severely tested, and in the context of a broader and more generic legislative reform. Judicial litigation can indeed be considered one of the main deterrents to choosing healthcare professions, both in Italy and in other states [21,22,23,24].

## Data Availability

Not applicable.
